# Lose-of-Function of a Rice Nucleolus-Localized Pentatricopeptide Repeat Protein Is Responsible for the *floury endosperm14* Mutant Phenotypes

**DOI:** 10.1186/s12284-019-0359-x

**Published:** 2019-12-30

**Authors:** Mengyao Xue, Linglong Liu, Yanfang Yu, Jianping Zhu, Hao Gao, Yihua Wang, Jianmin Wan

**Affiliations:** 1 State Key Laboratory for Crop Genetics and Germplasm Enhancement, Jiangsu Plant Gene Engineering Research Center, Nanjing Agricultural University, Nanjing, 210095 People’s Republic of China; 2 National Key Facility for Crop Resources and Genetic Improvement, Institute of Crop Science, Chinese Academy of Agricultural Sciences, Beijing, 100081 People’s Republic of China

**Keywords:** Rice (*Oryza sativa*), *flo14* mutant, *OsNPPR3*, PPR protein, Nucleolus-targeted

## Abstract

**Background:**

The endosperm of rice (*Oryza sativa*) has been usually used for the study of starch synthesis. Although several related factors have been revealed, other unknown members remain to be identified, given that starch synthesis is a complicated and sophisticated process.

**Results:**

Here, we identified and characterized a new rice seed mutant, *floury endosperm14* (*flo14*), which showed chalked endosperm and seed-lethal phenotypes. Map-based cloning indicated *FLO14* encodes a novel P-family PPR protein which contains ten PPR motifs. Afterwards the gene was named *OsNPPR3*. Subcellular localization showed OsNPPR3 was targeted to nucleolus. Quantitative RT-PCR analysis demonstrated that *OsNPPR3* was universally expressed in various tissues, with pronounced levels during rice endosperm development. Molecular analysis further suggested that *OsNPPR3* was involved in the regulation of expression levels and splicing of a few genes in mitochondria.

**Conclusion:**

The study demonstrates that the nucleolus-localized PPR protein is responsible for the *flo14* mutant phenotypes through affecting nuclear and mitochondrial gene expression and splicing.

## Background

Crop plants accumulate large amounts of starch in storage tissues, such as the endosperm in rice (*Oryza sativa*) and maize, as the main carbon sources for human and livestock (Burrell, 2003). Starch biosynthesis is major in the amyloplast, a kind of specialized plastid in the endosperm cell (Martin and Smith, 1995). Although some related factors involved in starch biosynthesis have been reported in rice (Long et al., 2017), there still existed a large number of unknown genes related to starch synthesis in rice. Floury endosperm (flo) mutants are ideal genetic materials for studying the mechanism of starch biosynthesis and amyloplast development.

The first floury endosperm mutant (*flo1*) was previously reported to locate on chromosome 5, and the causative gene was not yet identified (Satoh and Omura, 1981). The *flo2* locus was revealed to encode a nuclear-localized TPR-binding protein, which influenced starch synthesis potentially via interaction with transcription factors such as bHLHs to positively regulate expression of starch synthesis-associated genes (She et al., 2010). The *flo3* mutant showed floury endosperm, accompanied by a low level of the 16-kDa globulin (Nishio and Iida, 1993). The opaque endosperm mutant *flo4* was due to an insertional mutation in the *OsPPDKB* (*cytosolic pyruvate orthophosphate dikinase*) gene. Further study showed the *OsPPDKB/FLO4* gene could act as an important modulator of carbon flow for starch and lipid biosynthesis during grain filling (Kang et al., 2005). Later on, the *OsSSSIIIa* (*soluble starch synthase IIIa*) mutation was identified as the cause of *flo5* mutant. The OsSSSIIIa/FLO5 protein played an important role in generating relatively long chains in rice endosperm (Ryoo et al., 2007). Recently, a series of rice *flo* mutants were identified, including *flo6* (Peng et al., 2014)*, flo7*(Zhang et al., 2016)*, flo8* (Long et al., 2017)*, flo12* (Zhong et al., 2019)*,* and *flo15*(You et al., 2019). These mutants provided useful basis to understand the starch regulatory mechanism in rice.

Pentatricopeptide repeat (PPR) proteins are a large family of RNA-binding proteins in higher plants, with more than 600 members in Arabidopsis (*Arabidopsis thaliana*) (Cheng et al., 2016), and over 400 members in rice (Lurin et el., 2004; Gutie’rrez-Marcos et al., 2007). Increasing evidence indicates that PPR genes play an important role in plant post-transcriptional regulation, such as RNA editing (Takenaka, 2010; Barkan et al., 2012; Li et al., 2018), RNA splicing, RNA stability, RNA maturation, and translation initiation (Barkan et al., 2012). PPR proteins localize primarily in mitochondria and chloroplasts, and are essential for the normal biological activities of the both organelles (Zhang et al., 2017). Given only limited nucleus-localized PPR proteins have been identified in rice, the information of PPRs that regulate seed development and coordinate gene expression between the organelles and nucleus remains fragmentary.

In this study, we screened and obtained another *flo* mutant (*flo14*), which showed abnormal starch biosynthesis and seed-lethal phenotypes. Map-based cloning and complementation test revealed that *FLO14* locus encodes a nucleolus-targeted P-subfamily PPR protein, named OsNPPR3. Expression analysis indicated that the transcription and splicing of several nuclear-encoded and mitochondria-encoded genes were markedly altered in *flo14* relative to the wild type. Our results provide the first evidence that OsNPPR3 is involved in starch biosynthesis and seed vigor.

## Results

### Phenotypic Characterization of the *flo14* Mutant

**Fig. 1:**
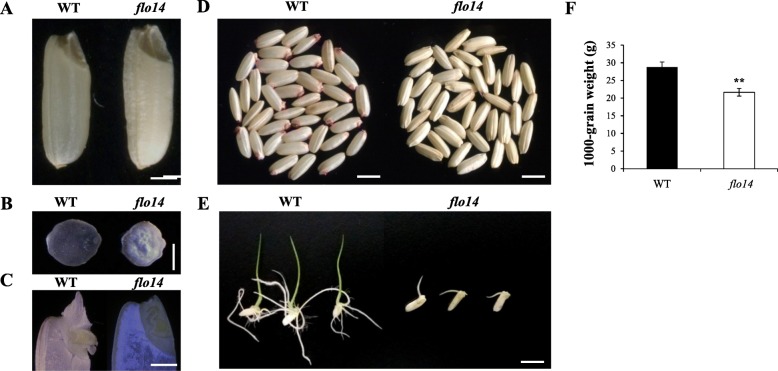
Phenotypic characterization of the *flo14* mutant. **a** Comparison of wild-type (WT) and *flo14* mutant (*flo14*) seeds. **b** Cross sections of wild-type and *flo14* mutant seeds. **c** Vertical-sections of imbibed embryos of wild type and *flo14* mutant. **d** Tetrazolium assay of wild-type and *flo14* mutant seeds. **e** Young seedlings of wild type and *flo14* mutant at 5 days after germination. **f** Thousand kernel weight of wild-type and *flo14* mutant seeds. Data indicate means ± SD (from at least three independent samples) and was compared with wild type by Student’s *t*-test (* *P* < 0.05, ** *P* < 0.01). Scale bars: 1 mm in (**a** and **b**), 1 cm in (**c** and **d**), 500 μm in (**e**)

A stably inherited mutant (named *flo14*) was obtained from a mutant library induced by N-methyl-N-nitrosourea. The mutant library was produced by the National Key Laboratory of Crop Genetics and Germplasm Enhancement, Nanjing Agricultural University. The *flo14* mutant was selected due to the chalky endosperm phenotype and aimed to study the function of starch-related genes. The *flo14* mutant was backcrossed twice with background parent to exclude the possibility of other gene variants, and the mutant seeds could only be collected from heterozygous individuals. At the mature stages, *flo14* mutant seeds showed floury endosperms in contrast to the transparent endosperm of wild type (Fig. 1a, b). Vertical-sections of imbibed seeds showed that wild-type embryos were well developed with established coleoptiles and shoot apical meristems, whereas only incomplete coleoptile structures were observed in the *flo14* embryos (Fig. 1c). The tetrazolium staining revealed that none of the *flo14* mutant seeds were stained red, indicating that the seed viability of the mutants was severely reduced (Fig. 1d). The seed germination test showed the *flo14* mutant produced no complete shoots and roots, and died about 10 days after germination (Fig. 1e), suggesting the embryogenesis of *flo14* was compromised. Consistent with the floury endosperms, thousand kernel weight of *flo14* seeds was 10% reduced relative to the wild type (Fig. 1f).

### Starch Granule Development Is Defected in *flo14* Mutant

**Fig. 2:**
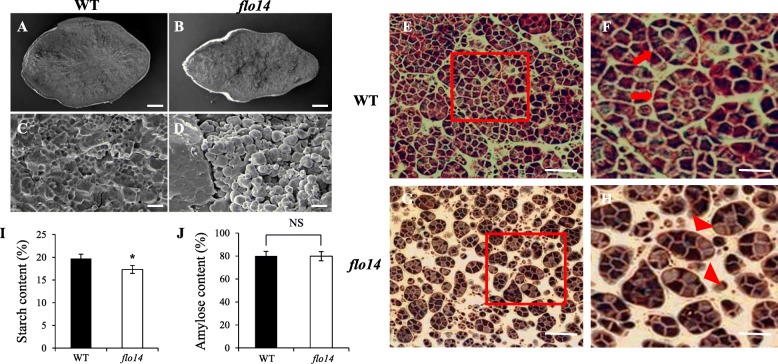
Starch granules development in the *flo14* mutant. **a**-**d** Scanning electron microscope (SEM) analysis of the endosperm in wild-type (WT) (**a**, **c**) and *flo14* mutant (*flo14*) (**b**, **d**) seeds. **e-h** Semi-thin sections of wild-type and *flo14* mutant seeds. The central part of 12 days after flowering (DAF) in the wild-type endosperm cells (**e-f**) and the central part of mutant *flo14* endosperm cells (**g-h**). **i** Starch content of wild type and *flo14* mutant (*n* = 3 each). **j** Amylose content of wild type and *flo14* mutant (*n* = 3 each). Data are shown as means ± SD (from at least three independent samples) and was compared with wild type by Student’s *t*-test (* *P* <0.05). NS: no obviously changed. Scale bars: 0.5 mm in (**a**, **b**), 15 μm in (**c**, **d**), 100 μm in (**e**, **g**), 200 μm in (**f**, **h**)

To determine the morphologic details of the mutant seeds, we performed scanning electron microscope (SEM) examinations. The results indicated that the starch granules of *flo14* mutant were loosely packed. In contrast, wild-type ones were equal-sized and densely arranged (Fig. 2a-d). Besides, semi-sectioning was conducted to observe starch granules in developing endosperm at 12 days after flowering (DAF). In the center of wild-type endosperm, the amyloplast was composed of several mature granules that were in large qualities and closely arranged (Fig. 2e, f, red arrowheads). Nevertheless, smaller, immature and more scattered starch granules were observed in the mutant, and they were separated from each other, and a great variety of gaps appeared in the cytoplasmic space (Fig. 2g, h, red triangular arrowheads).

In the *flo14* mutant, total starch content was significantly lower than in the wild type. Correspondingly, the amylose content was slightly reduced but not obviously (Fig. 2i, j). Meanwhile, we compared the swelling volume of wild-type and *flo14* mutant starch in different urea concentrations from 0 M to 9 M (Additional file 1: Figure S1a, b). We found wild-type starch began to dissolve in 4 M urea solution, while *flo14* mutant started to dissolve in 5 M urea. Furthermore, the pasting properties of endosperm starch were analyzed by rapid visco analyzer (RVA; Additional file 1: Figure S1c). The viscosity pattern of the *flo14* pasting starch was not obviously different from that of the wild type. Together, the results showed both the fine structure and physicochemical properties of the starch granules had been changed in the *flo14* mutant.

### Map-Based Cloning of the Gene Responsible for the *flo14* Phenotypes

Genetic analysis was conducted in the offspring derived from a single plant containing the heterozygous *flo14* gene. The numbers of seeds with normal (transparent endosperm and normal seed vigor) and mutant phenotypes (chalky endosperm and seed lethality) were counted separately. Normal: mutant seeds = 188: 67, χ^2^ = 0.212 < χ^2^_0.05,1_ = 3.842 (Additional file 2: Table S1), indicating that the mutant phenotypes is controlled by a single recessive gene.

**Fig. 3:**
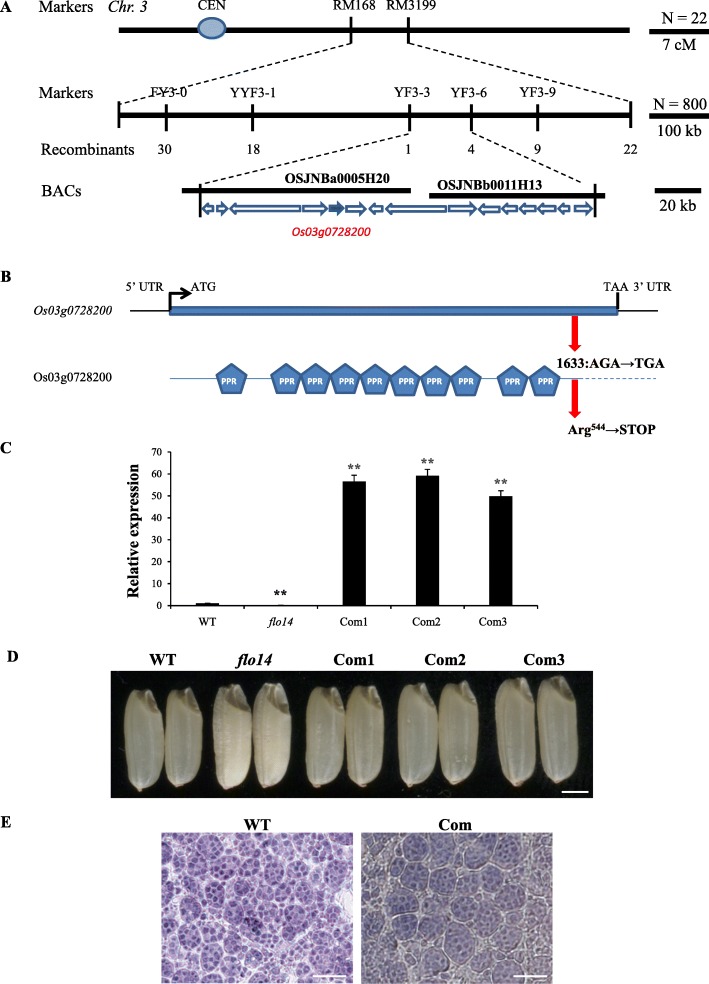
Map-based cloning of the gene responsible for the *OsNPPR3* phenotypes. **a** Fine mapping of the *OsNPPR3* locus. The *OsNPPR3* locus was mapped to a 165-Kb region by markers FY3–3 and FY3–6 on Chromosome 3 (*Chr.3*), which contains 15 predicted genes. The number of recombinants is indicated below the map. The candidate gene is indicated by red arrow. **b** The PPR gene structure and its protein. The lines indicate 5′-UTR and 3′-UTR, respectively. The blue box means exon. ATG and TAA represent start codon and stop codon. The PPR protein contains 10 PPR motifs. A single nucleotide substituted in the coding region of mutant gene leads to a premature stop codon. **c** Real-time RT-PCR analysis of *OsNPPR3* in developing endosperms at 12 days after flowering (DAF) in the wild type (WT), *flo14* mutant (*flo14*) and three *flo14* mutant lines expressing the wild-type *OsNPPR3* gene (Com1, Com2 and Com3). The value of *Actin I* mRNA was used as an internal control for data normalization. **d** Complementation of the *flo14* mutant restored normal seed appearance. **e** Semi-thin sections of wild type and the complementation of the *flo14* mutant (Com) endosperm at 15 days after flowering. Values are means ± SD (*n* = 3). The asterisks indicate statistical significance compared with the *flo14* mutant, as determined by a Student’s *t*–test (** *P* < 0.01). Scale bars: 1 mm in (**d**), 100 μm in (**e**)

To map the mutant gene locus, we generated an F_2_ population from a cross between the *flo14* mutant and *indica* cultivar N22. Ten individuals containing floury white endosperm were chosen from the F_2_ progeny, and the gene locus was roughly mapped on the long arm of chromosome 3 between the markers RM168 and RM3199. Then we used additional eight hundred recessive individuals from the same F_2_ population and narrowed it to a 165–kb region, which contained two Bacterial Artificial Chromosomes (BACs) and fifteen open reading frames (Fig. 3a). Comparison of the sequences between the wild type and *flo14* mutant revealed a single nucleotide replacement occurred at the only exon of *Os03g0728200*, leading to a premature termination of transcription at amino acid residue 1633 of a PPR protein. Afterwards the candidate gene was named *OsNPPR3*. The wild-type PPR protein was composed of 1806 amino acid residues and contained a tandem repeat of 10 PPR motifs, and was thus classified as P- subclass (Fig. 3b).

**Fig. 4:**
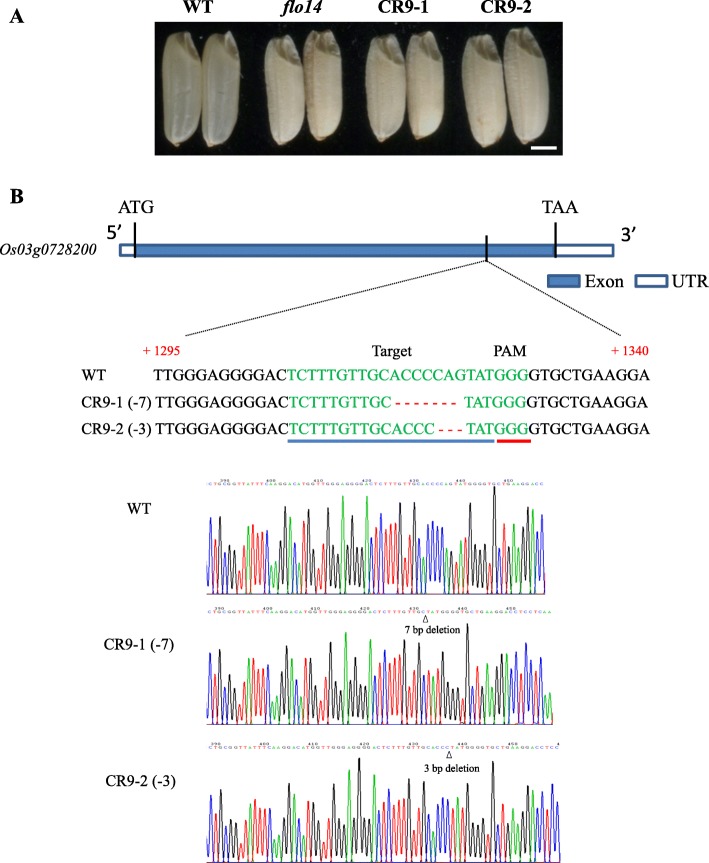
Knockout of the *OsNPPR3* gene by CRISPR system. **a** Seeds of the wild type (WT), *flo14* mutant (*flo14*), and two independent CRISPR/Cas9 T_1_ transgenic lines, which were named CR9–1 and CR9–2. **b** Verification of the knockout lines by PCR-based sequencing. The representative transgenic lines were generated from *Oryza sativa L. japonica* variety Dianjingyou1 genetic background. ATG and TAG indicate the start and stop codons, respectively. UTR means untranslated region. Arabic number indicates the base position from the start codon. The target sequences designed for knocking out the *Os03g0728200* by CRISPR/Cas9 system and the protospacer-adjacent motif (PAM) are underlined in blue and red, respectively. The missing bases are marked with red dotted lines. The chromatograms of the wild type and two knockout lines (CR9–1 and CR9–2) are shown. Triangle means the site of base deletion in the knockout line. Scale bars:1 mm in (**a**)

To verity *OsNPPR3* was responsible for the *flo14* mutant phenotypes, we cloned a wild-type genomic fragment including the entire *OsNPPR3* coding sequence into the pCUbi1390 binary vector, and then introduced the over-expression gene construct into the *flo14* mutant. The expression levels of positive transgenic complementary lines were significantly higher than the control in mature seed endosperms (Fig. 3c). The seeds harvested from the transgenic positive T_1_ lines (*FLO14* +/+) were transparent (Fig. 3d) and could germinate and grow normally (Additional file 3: Figure S2), indicating that the developmental defects of embryo and endosperm in *flo14* were completely rescued. Meanwhile, semi-thin sectioning indicated the starch granules of the transgenic complementary lines were also restored to the wild-type structure (Fig. 3e). Besides, *OsNPPR3* knockout plants generated by the CRISPR/Cas9 system exhibited similar phenotypes as the mutant (Fig. 4). Collectively, the results suggested the P-type PPR is the causative gene of the *flo14* mutant*.*

### Expression Pattern Analysis of *OsNPPR3* and Genes Associated with Starch Synthesis

**Fig. 5:**
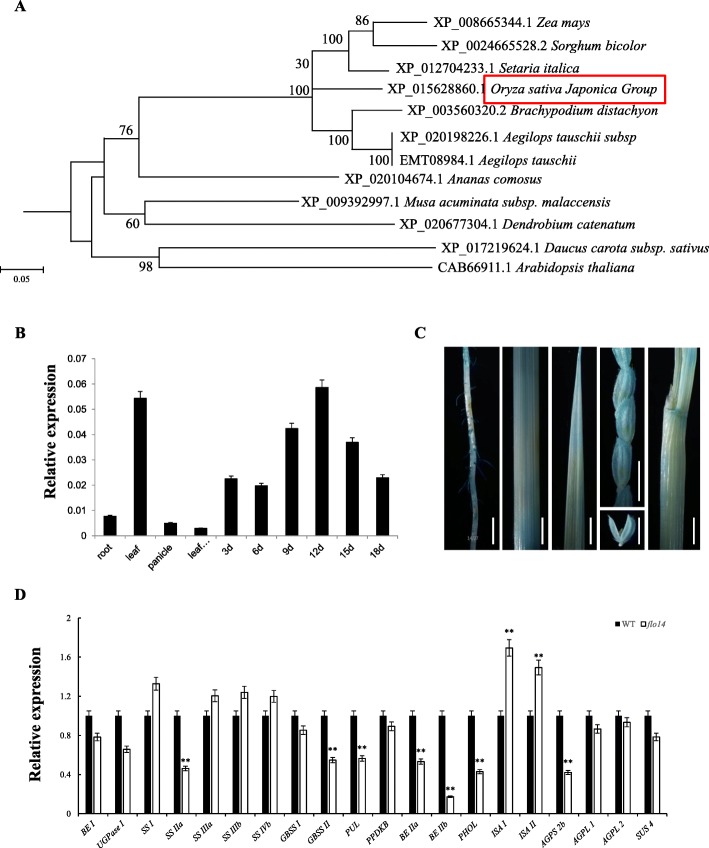
Homologous comparison and expression analysis. **a** A neighbor-joining tree of PPR gene and its homologs. The tree was constructed using MEGA and bootstrapped with 1000 replicates. The proteins are named according to their gene/EST names or NCBI accession numbers. *OsNPPR3* was indicated by the red frame. **b** Expression levels of PPR gene in various tissues and different developmental stages endosperm of the wild type (*n* = 3 each). **c** Histochemical staining showed that PPR: GUS reporter gene is ubiquitously expressed in the root, stem, leaves, panicles and leaf shoots from the left photo to the right, respectively. **d** Real-time PCR analysis of starch synthesis genes in 12 days after flowering (DAF) wild-type (WT) and *flo14* mutant (*flo14*) seeds. *Actin1* was used as an internal control. Data gives as means ± SD from three independent biological replicates and was compared by Student’s *t*-test (* *P* < 0.05, ** *P* < 0.01). Scale bars: 1 cm for all panels in (**c**). *BE I: branching enzyme I, UGPase I: UTP-glucose-1-phosphate-uridyly-1 transferase, SS I:soluble starch synthase I, SS IIa: soluble starch synthase IIa, SS IIIa: soluble starch synthase IIIa, SS IIIb: soluble starch synthase IIIb, SS IVb: soluble starch synthase IVb, GBSS I: granule-bound starch synthase I, GBSS II: granule-bound starch synthase II, PUL: pullulanase, PPDKB: pyruvate phosphate dikinase B, BE IIa: branching enzyme IIa, BE IIb: branching enzyme IIb, PHOL: starch phosphorylase L, ISA I: Isoamylase I, ISA II: Isoamylase II, AGPS 2b: ADP-glucose pyrophosphorylase 2b, AGPL 1: ADP-glucose pyrophosphorylase large subunit 1, AGPL 2: ADP-glucose pyrophosphorylase large subunit 2, SUS 4: sucrose synthase 4*

Phylogenetic analysis indicated *OsNPPR3* shared a high similarity with its homologs in other plants, including *Zea mays, Sorghum bicolor, Setaria italic, Brachypodium distachyon, Aegilops tauschii subsp and Ananas comosus* (Fig. 5a, Additional file 4: Figure S3). At the same time, temporal and spatial expression analyses showed that *OsNPPR3* was universally expressed in various tissues (including roots, panicles, leaves and leaf sheathes) with the highest level in leaves. *OsNPPR3* transcripts were accumulated gradually during endosperm development and peaked at 12 DAF (Fig. 5b). To confirm the results, we transformed a vector with the GUS reporter gene driven by the *OsNPPR3* promoter into the rice. Histochemical analysis of GUS activity in independent transgenic plants corroborated that*OsNPPR3* presented a constitutive expression pattern (Fig. 5c).

To determine whether the defective *OsNPPR3* affected starch biosynthesis, we examined the expression profiles of major endosperm starch synthesis-related genes in developing endosperm by quantitative RT-PCR (qRT-PCR). Compared to the wild type, the expression levels of the tested genes were mostly changed in *flo14* mutant. Remarkably, the expression levels of *SS IIa, BE IIa*, *BE IIb*, *PHOL*, and *AGPS 2b* were significantly down-regulated, whereas *ISA I* and *ISA II* was obviously up-regulated in the *flo14* mutant (Fig. 5d). These results indicated that the starch accumulation was largely affected during endosperm development in the *flo14* mutant.

### Subcellular Localization of OsNPPR3

**Fig. 6:**
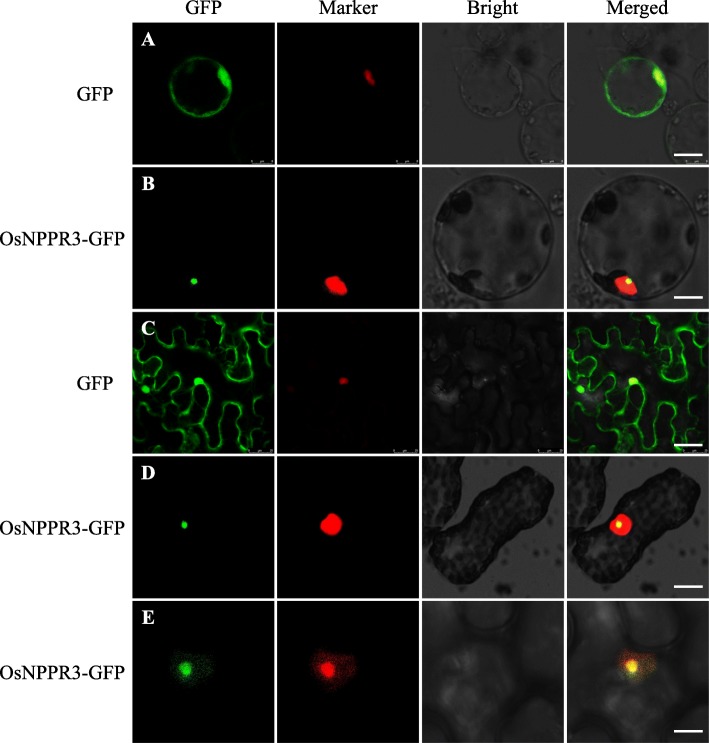
Subcellular localization of OsNPPR3. **a**-**e** Transient expression of 35S: OsNPPR3-GFP fusion protein located in the nucleolus of rice protoplasts (**a**, **b**) and *Nicotiana tabacum* protoplasts (**c**-**e**). Nucleus marker was used as a nucleus indicator (**a**-**d**). Nucleolus marker was used as a nucleolus indicator (**e**). RPBF (Rice Prolamin Box Binding Factor) and RPL23aB (r-Protein family member) were employed as nucleus-targeted and nucleolus-targeted marker, respectively. Scale bars: 1.6 μm in (**a**), 5 μm in (**b**, **c**), 10 μm in (**d**, **e**)

OsNPPR3 was predicted as a nucleus-targeted protein by the online tools TargetP (http://www.cbs.dtu.dk/services/TargetP/). To experimentally identify the predicted subcellular localization and the length and position of the nucleus-targeting signal in OsNPPR3 protein, a green fluorescent protein (GFP) fusion construct driven by the cauliflower mosaic virus *35S* (CaMV-*35S*) promoter was generated, which contained a full-length OsNPPR3 coding region in front of the GFP protein and was named as OsNPPR3-GFP. Meanwhile, RPBF (Rice Prolamin Box Binding Factor) was employed as a nucleus-localized marker (Kawakatsu et al., 2009) and RPL23aB (r-Protein family member) as a nucleolus-targeted marker (Degenhardt and Bonham-Smith, 2008). All constructs were transiently expressed in both rice protoplasts (Fig. 6a, b) and tobacco leaves (Fig. 6c-e). The free GFP signals were diffused in the cytoplasm (Fig. 6a, c). By contrast, OsNPPR3-GFP fusion protein was localized in nucleus (Fig. 6b, d), and more accurately, in nucleolus because the signals were more similar to RPL23aB than RPBF (Fig. 6e). These results confirmed that OsNPPR3 protein is targeted to the nucleolus.

### *OsNPPR3* Affects Expression of Mitochondrial Genome-Encoded Genes and Mitochondrial Ultrastructure

**Fig. 7:**
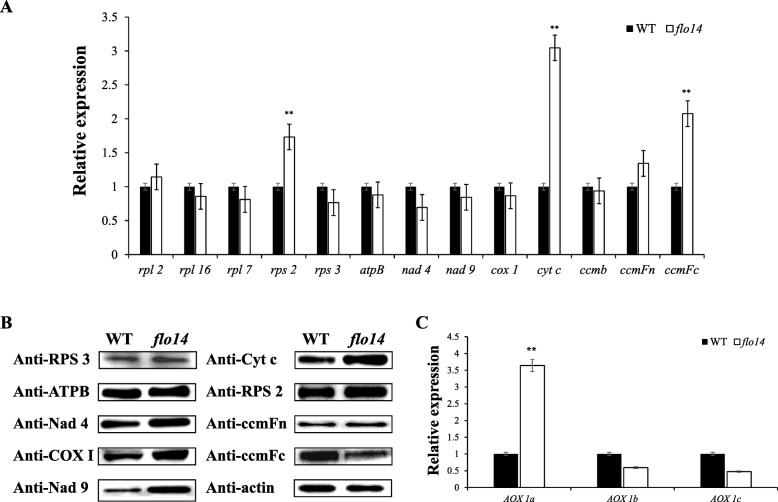
Expression analysis of *OsNPPR3* and genes associated with mitochondria. **a** Real-time RT-PCR analysis of the mitochondrial gene expression in endosperms of wild type (WT) and *flo14* mutant (*flo14*) at 12 days after flowering (DAF). **b** Immunoblotting analysis of mitochondria related proteins in developing endosperm of wild type and *flo14* mutant. **c** Real-time RT-PCR analysis of the gene expression in the wild type and *flo14* mutant. Data are shown as means ± SD from three independent biological replicates and compared by Student’s *t*-test (* *P* < 0.05, ** *P* < 0.01). *Actin1* was used as an internal control. *rpl2*: *ribosomal protein L2*, *rpl16*: *ribosomal protein L16*, *rpl7*: *ribosomal protein L7*, *rps2*: *ribosomal protein S2*, *rps13*: *ribosomal protein S13*, *cox1*: *cytochrome c oxidase subunit 1*, *cyt c*: *cytochrome C*, *ccmb: cytochrome c biogenesis B*, *ccmFn*: *cytochrome c biogenesis Fn*, *ccmFc: cytochrome c biogenesis Fc*, *nad4*: *NADH dehydrogenase subunit IV*, *nad9*: *NADH dehydrogenase subunit XI*, *AOX 1a*: *alternative oxidase 1a*, *AOX 1b*: *alternative oxidase 1b*, *AOX 1c*: *alternative oxidase 1c*

PPR proteins are important for mitochondrial-encoded protein synthesis and mitochondrial function (Liu et al., 2013). The seed lethal phenotype of homozygous mutant suggested that the *flo14* mutant might have defects in gene expression relating to mitochondria functions. For validation, expression analysis performed by qRT-PCR using RNA samples prepared from 12 DAF endosperms. Of 13 mitochondrial genome-encoded electron transport chain genes, three (including *rps2, cyt c* and *ccmFc*) were significantly up-regulated, while the others showed no difference between the *flo14* mutant and its wild type (Fig. 7a). Further, mitochondrial electron transport chain (ETC) complex proteins were isolated from developing endosperm and analyzed by western blotting. The *flo14* mutant displayed an increase abundance of both the rps2 and cyt c bands, but a slight decrease in the ccmFc band compared to the wild-type profile (Fig. 7b), indicating that the accumulation of ETC components is disturbed in the *flo14* mutant.

When an electron transport is defective in the cytochrome c pathway, alternative oxidases (AOXs) are generally activated to maintain the tricarboxylic acid cycle and electron transport (Vanlerberghe and Ordog, 2002). The expression of *AOX* genes in the *flo14* mutant showed that an *AOX* (*AOX 1a*) was up-regulated 5-fold, whereas other two *AOXs* (*AOX 1b* and *AOX 1c*) demonstrated no significant change relative to the wild type (Fig. 7c), suggesting that the alternative respiratory pathway was activated to compensate for the mitochondrial dysfunction in the *flo14* mutant.

**Fig. 8:**
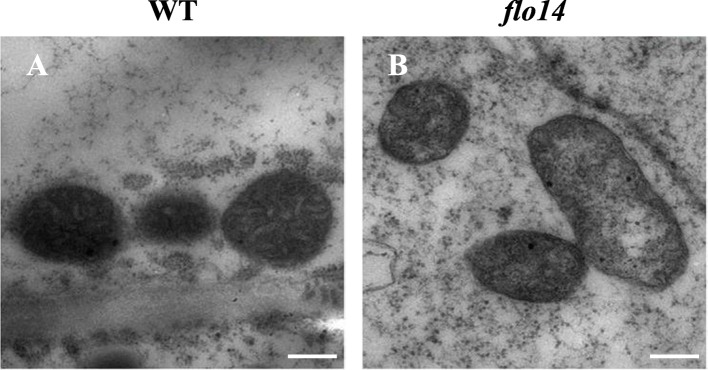
Transmission electron micrographs of mitochondria. Transmission electron micrographs of mitochondria from 12 days after flowering (DAF) wild-type (WT) (**a**) and *flo14* mutant (*flo14*) (**b**) endosperms. Scale bars: 1 μm in (**a**) and (**b**)

Activation of the ETC is essential for the proper formation of the inner envelope cristae in mitochondria (Logan, 2006). Next, we performed transmission electron microscopic analysis to detect any morphological changes in mitochondria of developing endosperm cells. In contrast to the wild-type mitochondria that formed clear inner envelope cristae surrounded by a dense matrix, a large portion of mutant mitochondria lacked obvious cristae internal structure and the mitochondria matrix was extremely porous. Besides, the appearance of the mutant mitochondria seemed irregular and more dilated than the wild type (Fig. 8).

### The *flo14* Mutant Is Defective in the Splicing of Mitochondrial Genome-Encoded Genes

**Fig. 9:**
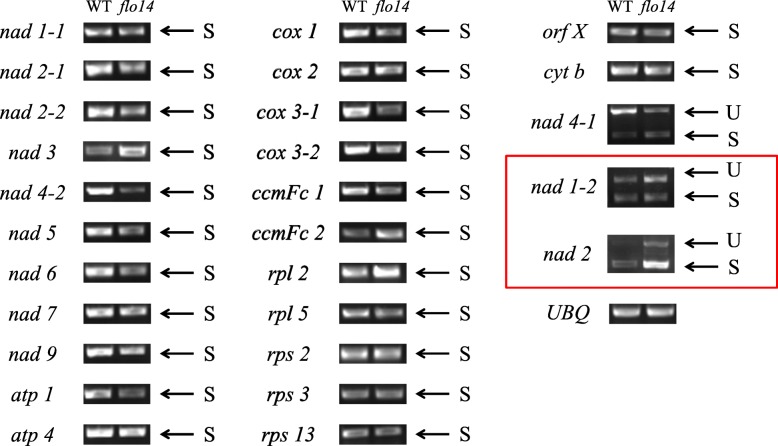
The *flo14* mutant is defective in the splicing of mitochondria genes. Gene transcripts are labeled at the left. Spliced (S) and unspliced (U) transcripts are shown at the right. RNA were extracted from 12 days after flowering (DAF) endosperms of wild type (WT) and *flo14* mutant (*flo14*). The splicing genes are tagged with red boxes. *UBQ* was used as a quantitative control. *Nad 1–1: NADH dehydrogenase subunit 1–1, nad 1–2: NADH dehydrogenase subunit 1–2, nad 2: NADH dehydrogenase subunit 2, nad 2–1: NADH dehydrogenase subunit 2–1, nad 2–2: NADH dehydrogenase subunit 2–2, nad 3: NADH dehydrogenase subunit 3, nad 4–1: NADH dehydrogenase subunit 4–1, nad 4–2: NADH dehydrogenase subunit 4–2, nad 5: NADH dehydrogenase subunit 5, nad 6: NADH dehydrogenase subunit 6, nad 7: NADH dehydrogenase subunit 7, nad 9: NADH dehydrogenase subunit 9, atp 1: ATP synthase F0 subunit 1, atp 4: ATP synthase F0 subunit 4, cox1: cytochrome c oxidase subunit 1, cox 2: cytochrome c oxidase subunit II, cox 3–1: cytochrome c oxidase subunit 3–1, cox 3–2: cytochrome c oxidase subunit 3–2, ccmFc: cytochrome c biogenesis Fc (two pairs of primers were used), rpl 2: 50S ribosomal protein L2, rpl 5: ribosomal protein L5, rps 2: 30S ribosomal protein S2, rps 3: 30S ribosomal protein S3, rps 13: ribosomal protein S13, orf X: hypothetical protein, cyt b: cytochrome b*

Accumulating evidence shows that a large group of nuclear encoded PPR proteins were required for RNA splicing, editing, stability, maturation, and translation (Pfalz et al., 2009; Sosso et al., 2012; Li et al., 2014; Xiu et al., 2016; Wang et al., 2017). To determine whether the *flo14* mutation affected RNA splicing of mitochondria genes, we carried out RT-PCR analysis using primers spanning exons of 27 mitochondrial genome-encoded transcripts and compared the lengths of the amplified products between the wild type and *flo14* mutant plants. We found two transcripts, including *nad 1–2* and *nad 2*, were spliced with much reduced efficiency in the *flo14* mutant compared to the wild type. In contrast, splicing of the other transcripts showed no significant difference between the *flo14* mutant and its wild type (Fig. 9). This result illustrated that the *flo14* mutation partially affected splicing of mitochondrial genome-encoded transcripts.

## Discussion

Endosperm is a storage organ in which a massive amount of storage starch and storage proteins are accumulated during seed development (She et al., 2010). In rice, several key enzymes involved in starch biosynthesis have been well studied, including AGPase (ADP glucose pyrophosphorylase) (Muller-Rober et al., 1992), GBSS (granule-bound starch synthase) (Caballero et al., 2008), SS (soluble starch synthase) (Hirose and Terao, 2004), and starch branching enzyme (BE) (Mizuno et al., 1993). Through a series of endosperm defective mutants, such as floury, white-core or opaque kernel (Satoh and Omura, 1981), several regulatory genes associated with starch synthesis have been isolated. For example, *FLO6* encodes a CBM containing protein, and its mutant showed decreased starch content and altered starch physicochemical features (Peng et al., 2014). *FLO12* encodes an alanine aminotransferase, and the *flo12* mutant exhibited loosely packed starch granules and a lower thousand kernel weight compared to its wild type. Semi-thin sections revealed that compound starch grains in *flo12* interior endosperm cells were developed abnormally and amylose content was also decreased (Zhong et el., 2019). Besides, the rice *FL*O15 encodes a glyoxalase I. A growth arrest of the *flo15* mutant amyloplasts was observed during the early grain-filling stage, causing the generation of abnormal amyloplasts lacking compound starch grains (You et al., 2019). In our study, the *flo14* mutant showed a similar chalky endosperm phenotype to the former endosperm mutants (Fig. 1). The SEM and semi-sectioning results showed that starch granules in the grains of the mutant were compromised (Fig. 2a-h). Meanwhile, total starch content of *flo14* mutant was significantly lower than that in the wild type and the amylose content was slightly reduced (Fig. 2i, j). These results indicated that mutation of *flo14* dramatically affects starch synthesis in rice endosperm.

PPR is a large protein family implicated in organellar gene expression, including RNA editing, intron splicing, RNA processing, RNA maturation, RNA stability, and translation initiation (Rivals et al., 2006). Previous studies isolated and characterized numerous PPR genes such as *THYLAKOID ASSEMBLY 8* (Khrouchtchova et al., 2012), *PPR4* (Schmitz-Linneweber et al., 2006) and *PPR5* (Beick et al., 2008), and they were required of splicing of *ycf3–2*, *rps12* and trnG-UCC precursor, respectively. By map-based cloning, we found that the single nucleotide replacement occurred at the exon of *Os03g0728200*, leading to a premature termination of transcription (Fig. 3a, b). *Os03g0728200* encoded a P-type subfamily PPR protein with ten PPR motifs and its function was not yet revealed before. Seed harvested from independent complementary positive T_1_ lines showed similar phenotypes to the wild type (Fig. 3d, e), whereas lose-of-function of *OsNPPR3* by CRISPR technology mimicked the mutant grain phenotypes (including the chalky endosperm and seed lethality). Considering the mutagenesis sites were near to the start codon, the protein of CRISPR/Cas9 mutant was supposed to be functionally missed in the plant. Meanwhile, little transcript was detected in the *flo14* mutant. The results suggested the *flo14* mutant might also carry a functionally defective OsNPPR3 protein, just like the CRISPR/Cas9 mutants. Collectively, these results strongly supported that *OsNPPR3* plays a curial role in starch granules development and seed vigor in rice.

Up to now, only limited PPR proteins have been reported to localize in nucleus or nucleolus. In *Arabidopsis thaliana*, GRP23 (Glutamine-Rich Protein 23) is a nucleus-localized PPR protein that functions as a potential regulator of gene expression during early embryogenesis (Ding et al., 2006). Loss-of-function mutation of GRP23 causes the arrest of early embryo development. Besides, another PPR (PNM1, for PPR protein localized to the nucleus and mitochondria 1) was reported to dual localize to the nucleus and mitochondria. PNM1 acts as a retrograde messenger molecule from mitochondria to the nucleus for the fine-tuning of nuclear gene expression, which is required for mitochondrial biogenesis (Hammani et al., 2011). Recently, another PPR protein named OsNPPR1 [encoding by *FLOURY AND GROWTH RETARDATION1*(*FGR1*) locus] was revealed to affect splicing of six nuclear genes, many of which were related to mitochondrial functions (Hao et al., 2019). Similar to *flo14*, the *osnppr1*/*fgr1* mutant produced an opaque grain appearance with loosely arranged starch grains, decreased starch and amylose contents in endosperm. Likewise, *osnppr1* also displayed reduced grain weight and weaker seed germinated vigor relative to its wild type. However, the seedlings from homologues *osnppr1* seeds gradually recovered and grew into normal adult plants. Although the *osnppr1* plants exhibited significantly slower grain filling after fertilization, they could produce fertile seeds. In addition, OsNPPR1 was revealed to be nucleus-localized (Hao et al., 2019), instead of nucleolus-localization as shown in OsNPPR3 (Fig. 6). These results suggested OsNPPR1 and OsNPPR3 might play divergent functions for seed development.

Loss of OsNPPR3 function impairs mitochondria structure and is lethal for the embryo. Mitochondria are semi-autonomous organelles that have arisen from an endosymbiotic event (Gray et al., 2001). Although mitochondria have a complete gene expression machinery, the vast majority of mitochondrial proteins are encoded in the nucleus and have to be imported into mitochondria from the cytosol (Burger et al., 2003). Thus, mitochondrial biogenesis relies heavily on the coordinated expression of nuclear encoded genes (Hammani et al., 2011). Meanwhile, mitochondria are cytoplasmic organelles functioning as the factory for energy processing. Seed-lethal and chalky endosperm (a kind of poor grain filling) phenotypes implied some problems might happen in cell energy provision. In addition, the *flo14* phenotypes mutant was very similar to rice *ogr1* (o*paque and growth retardation1*) mutant with defective PPR protein *(*Kim et al., 2009)*.* Moreover, a number of other PPR proteins were reported to tightly related to mitochondrial function, including *RF5* (R*ESTORER-OF FERTILITY GENE5,* Hu et al., 2006) and *RF6* (R*ESTORER-OF FERTILITY GENE6,* Huang et al., 2015) in rice, as well as *DEK37* (*DEFECTIVE KERNEL 37*) in maize (Dai et al., 2018). These studies promoted us to study mitochondrial genes in *flo14*. We found the expressions of three mitochondrial-genome encoded genes were significantly altered in the *flo14* mutant (Fig. 7a). These results suggested that mitochondrion genes might be impaired in the mutant due to the mutation of *OsNPPR3* gene. Similar phenotypes were observed in *PpPPR_71* mutant, with the gene encoded a polypeptide of 833 amino acid with 17 PPR motifs and the C-terminal DYW domain (Tasaki et al., 2010). In the *flo14* mutant, both *rps2* and *Cyt c* gene expressions and their encoded proteins were consistently up-regulated. Intriguingly, the gene expression level of *ccmFc* was down-regulated, while its protein abundance was obviously accumulated compared with the wild type (Fig. 7b). This discrepancy may be due to a feedback regulation or post-transcriptional modification.

RNA splicing is a process of removing introns between neighboring exons during translation (Wu et al., 2012). Several RNA binding factors have important roles in RNA splicing in higher plants including PPR proteins and other nuclear-encoded factors (de Longevialle et al., 2010). A small number of PPR proteins have been discovered to be involved in RNA splicing in mitochondria. In maize, *dek2* (*defective kernel 2*) is a classic mutant with small kernels and delayed development (Qi et al., 2016). Mitochondrial transcript analysis indicated that *dek2* mutation caused reduced splicing efficiency of mitochondrial *nad1* intron 1. Mitochondrial complex analysis in *dek2* immature kernels showed severe deficiency of complex I assembly. *EMP10* (*EMPTY PERICARP 10*) is specifically required for the *cis*-splicing of mitochondrial *nad2* intron 1, embryogenesis and endosperm development (Cai et al., 2017). Likewise, *DEK37*s involves in *cis*-splicing of mitochondrial *nad2* intron 1 and is required for mitochondrial function and seed development in maize (Dai et al., 2018). In our study, the *flo14* mutant caused the defects in the splicing of *nad 1–2* and *nad 2* (Fig. 9), similar to the abovementioned mutants. The NAD 1 and NAD 2 are two of the nine complex I proteins encoded by the mitochondria genome (Brandt, 2006). The splicing defect of certain *nad* mRNAs was reported to result in partial or complete loss of ETC complex I activity as well as an obvious disturbance of growth and development (Cai et al., 2017). It is plausible that OsNPPR3 might be associated with the fine-tuning of transcript levels in the nucleus for mitochondrial biogenesis. Further studies will be needed to address this hypothesis in the future.

## Conclusions

We identified a mutant of *flo14*, which exhibited a floury endosperm and embryo-lethal phenotypes. Map-based cloning revealed that *OsNPPR3* encodes a nucleolus-localized P-family PPR protein that was validated by transgenic complementation and CRISPR experiments. The role of *OsNPPR3* in rice seed development was tightly related with mitochondrial ultrastructure as well as RNA splicing of mitochondrial genome-encoded genes. Our research results provide useful information for the future research to uncover the regulatory mechanism of PPR proteins during rice seed development.

## Methods

### Plant Material and Growth Conditions

The *flo14* mutant was selected from a chemical mutagenic bank of *japonica* variety Dianjingyou1. An F_2_ population was produced from a cross between the *flo14* mutant and *indica* cultivar N22 for mapping. The mutagenic bank was produced by the National Key Laboratory of Crop Genetics and Germplasm Enhancement, Nanjing Agricultural University. Plants were grown under natural and greenhouse conditions.

### Scanning Electron Microscope

Wild-type and *flo14* mutant seeds were transversely cut and examined with a HITACHI S–3400 N scanning electron microscope (http://www.hitachi-hitec.com). Scanning electron microscopy was performed as described in the previous article (Kang et al., 2005).

### Light Microscope

For the observation of compound granules, transverse sections of developing endosperms (approximately 1 mm in thickness) from the wild-type and *flo14* mutant seeds were fixed overnight in 0.1 M phosphate buffer (pH 7.2) with 2% (v/v) glutaraldehyde and 2% (w/v) paraformaldehyde. After dehydration in an ethanol series, samples were embedded in LR White resin (London Resin, Berkshire, UK, http://www.2spi.com/), followed by sectioning with an ultramicrotome (Leica UC7; http://www.leica-microsystems.com). Semi-thin sections were stained with I_2_-KI for 5 s and subsequently examined under a light microscope (80i, Nikon, http://www.nikon.com).

### Transmission Electron Microscope

Wild-type and *flo14* mutant endosperms samples were obtained from 12 DAF developing plants, which were grown under natural conditions. Endosperm sections were fixed in 2.5% glutaraldehyde in a phosphate buffer at 4 °C for 4 h, then washed and incubated in 1% OsO_4_ at 4 °C for 12 h. After dehydration in a gradient ethanol series, samples were embedded in Spurr’s resin prior to ultrathin sectioning. Sections were stained with uranyl acetate and examined with a Hitachi-H7650 transmission electron microscope.

### Analysis of the Starch Characters of Endosperm

The starch content of the rice flour was measured with a starch assay kit (Megazyme, Wicklow, Ireland, http://www.megazyme.com/), according to the manufacturer’s protocol. Amylose content was assessed following the method described by Liu et al. (2009).

### Mapping Cloning of *flo14*

To identify markers linked to *OsNPPR3*, we used *flo14* mutant, N22, 10 F_2_ plants with homozygous mutant alleles and 100 polymorphic SSR markers. *OsNPPR3* was mapped to a 165-Kb region with newly developed markers (Additional file 5: Table S2). We obtained the genomic sequences of candidate gene *Os03g0728200* from both the mutant and wild-type genome DNAs.

### Complementation Test

We cloned a wild-type genomic fragment including a 1.5 kb native promoter and entire *OsNPPR3* coding sequence into the pCUbi1390 binary vector, and then introduced the complementary gene construct into the *flo14* mutant *calli*. The primers were listed in Additional file 6: Table S3. The transgenic plants were grown in a greenhouse.

### CRISPR/Cas9 System

One target site that was 20 bp upstream of the protospacer-adjacent motif sequence (PAM) was designed for *Os03g0728200.* The combined target sequences were introduced to the gRNA-U3 and gRNA-U6 vectors, followed by two rounds of nested PCR with primers listed in Additional file 6: Table S3. The PCR products were subsequently linked to a CRISPR/Cas9 vector, and the whole construction was verified by PCR and sequencing. *Calli* derived from *japonica* variety Dianjingyou1 were used for *Agrobacterium*-mediated transformation. The original chromatogram files have been shown in Additional files 7, 8 and 9: Chromatograms S1 to S3. The knockout lines were grown in the greenhouse.

### Total RNA Extraction and Real-Time RT-PCR Analysis

Total RNA was extracted using an RNA prep pure Plant kit (TIANGEN Biotech, Beijing, China, http://www.tiangen.com/en/). A 1-μg portion of total RNA was reverse-transcribed by priming with oligo (dT_18_) in a 20 μg reaction based on the Prime Script Reverse Transcriptase kit (TaKaRa, http://www.takara-bio.com).The value of *ActinI* mRNA (accession number AK100267) was used as an internal control. The primers were listed in Additional file 10: Table S4 for detecting starch synthesis-related genes, Additional file 11: Table S5 for genes in mitochondria, and Additional file 12: Table S6 for genes associated with splicing in mitochondria.

### Histochemical Staining of GUS Expression

For GUS-staining assay, plant tissues were cut into small segments of approximately 2 cm^2^. GUS-staining assay was performed as described by Jeon et al. (Jeon et al., 2000). After staining, all samples were fixed in formalin-alcohol-acetic acid (FAA) fixative solution and embedded in paraffin (Kang et al., 2002). The samples were sectioned to a 12 thickness and observed with the microscope under dark-field illumination.

### Subcellular Localization

The *OsNPPR3* gene sequences were cloned in frame in front of the GFP coding region in the PAN580-GFP vector and pCMBIA1305 binary vector to create OsNPPR3-GFPs under the control of the CaMV *35S* promoters. The constructs were introduced into rice protoplasts separately and *Nicotiana tabacum* protoplasts. GFP fluorescent signals were detected using a confocal laser scanning microscope (Zeiss LSM710). The methods refer to the literature reported before (Chen et al., 2006).

### Protein Extraction and Western Blotting Analysis

Proteins were extracted from 0.5 g samples of developing seeds (12 DAF). Tissues were separately homogenized with a mortar and pestle in an extraction buffer of 100 mM Tris–HCl (pH 7.0), 2% (w/v) SDS, 2% (v/v) 2-mercaptoethanol, and 10% (v/v) glycerol. The resultant extracts were heated at 100 °C for 5 min, then centrifuged at 13,000 g for 20 min, and then recover the supernatants. The proteins were fractionated by SDS-PAGE and electro-blotted onto a nitrocellulose membrane using a buffer system consisting of 25 mM Tris-base, 144 mM glycine, 20% (v/v) methanol, and 0.1% (w/v) SDS. The membrane was incubated with a rabbit anti OsNPPR3 serum. OsNPPR3 was then detected on X-ray film using an HRP-conjugated mice anti rabbit IgG secondary antibody and ECL western blotting detection reagents according to the manufacturer’s protocol (Kang et al., 2005).

### Statistical Analysis

All statistical analyses were conducted using Excel 2010 (Microsoft Corp., Redmond, WA, USA).

## Supplementary information

**Additional file 1: Figure S1.** Comparison of starch physicochemical features.

**Additional file 2: Table S1.** The genetic analysis of *flo14* mutant.

**Additional file 3: Figure S2.** Young seedlings of wild type and the complementation of the *flo14* mutant.

**Additional file 4: Figure S3.** Comparison of amino acid sequences of the Os03g0728200 protein with its close species.

**Additional file 5: Table S2.** Markers used for *OsNPPR3* mapping in this study.

**Additional file 6: Table S3.** Primers used for vector construction.

**Additional file 7: Chromatogram S1.** The chromatogram for the wild type of target site.

**Additional file 8: Chromatogram S2.** The chromatogram for the transgenic line CR9–1 of target site.

**Additional file 9: Chromatogram S3.** The chromatogram for the transgenic line CR9–2 of target site.

**Additional file 10: Table S4.** Primers used for gene expression associated with starch synthesis.

**Additional file 11: Table S5.** Primers for gene expression in mitochondria.

**Additional file 12: Table S6.** Primers for genes associated with splicing in mitochondria.

## Data Availability

The materials used and/or analyzed during the current study are available from the corresponding author on request.

## Abbreviations

AGPase: ADP glucose pyrophosphorylase
*AGPL 1*: *ADP-glucose pyrophosphorylase large subunit 1*
*AGPL 2*: *ADP-glucose pyrophosphorylase large subunit 2*
*AGPS 2b*: *ADP-glucose pyrophosphorylase 2b*
*AOX 1a*: *Alternative oxidase 1a*
*AOX 1b*: *Alternative oxidase 1b*
*AOX 1c*: *Alternative oxidase 1c*
AOXs: Alternative oxidases
*atp 1*: *ATP synthase F0 subunit 1*
*atp 4*: *ATP synthase F0 subunit 4*
BAC: Bacterial artificial chromosomes
*BE IIa*: *Branching enzyme IIa*
*BE IIb*: *Branching enzyme IIb*
BE: Starch branching enzyme
CaMV-*35S*: Cauliflower mosaic virus *35S*
*ccmb*: *cytochrome c biogenesis B*
*ccmFc*: *cytochrome c biogenesis Fc*
*ccmFn*: *cytochrome c biogenesis Fn*
*cox 3–1*: *cytochrome c oxidase subunit 3-*
*cox 3–2*: *cytochrome c oxidase subunit 3–2*
*cox1*: *cytochrome c oxidase subunit 1*
*cox2*: *cytochrome c oxidase subunit 2*
*cty c*: *cytochrome c*
*cyt b*: *cytochrome b*
DAF: Days after flowering
*DEK2*: *DEFECTIVE KERNEL2*
*DEK37*: *DEFECTIVE KERNEL37*
*EMP10*: *EMPTY PERICARP 10*
ETC: Electron transport chain
flo: floury endosperm
GBSS: Granule-bound starch synthase
GFP: Green fluorescent protein
GRP23: Glutamine-Rich Protein 23
*ISA I*: *Isoamylase I*
*ISA II*: *Isoamylase II*
MNU: N-methyl-N-nitrosourea
*nad*: *NADH dehydrogenase subunit*
*ogr1*: o*paque and growth retardation1*
*orf X*: *hypothetical protein*
*OsPPDKB*: *Cytosolic pyruvate orthophosphate dikinase*
PAM: Protospacer-adjacent motif
*PHOL*: *Starch phosphorylase L*
PNM1: PPR protein localized to the nucleus and mitochondria 1
*PPDKB*: *Pyruvate phosphate dikinase B*
PPR: Pentatricopeptide repeat
*PUL*: *Pullulanase*
qRT-PCR: quantitative RT-PCR
*RF5*: *RESTORER-OF FERTILITY GENE 5*
*RF6*: *RESTORER-OF FERTILITY GENE 6*
RPBF: Rice Prolamin Box Binding Factor
*rpl*: *ribosomal protein*
RVA: Rapid visco analyzer
SEM: Scanning electron microscope
SS: Soluble starch synthase
*SUS*: *4* *Sucrose synthase 4*
*UGPase I*: *UTP-glucose-1-phosphate-uridyly-1 transferase*
WT: Wild type
