# Supplementary material for: Lose-of-Function of a Rice Nucleolus-Localized Pentatricopeptide Repeat Protein Is Responsible for the floury endosperm14 Mutant Phenotypes
Source: Rice (N Y). 2019 Dec 30;12:100. doi: 10.1186/s12284-019-0359-x (PMC6937366; doi:10.1186/s12284-019-0359-x)
Supplement: Supplementary file 1 — Additional file 1: Figure S1. Comparison of starch physicochemical features. [file 12284_2019_359_MOESM1_ESM.pptx]

## Slide 1
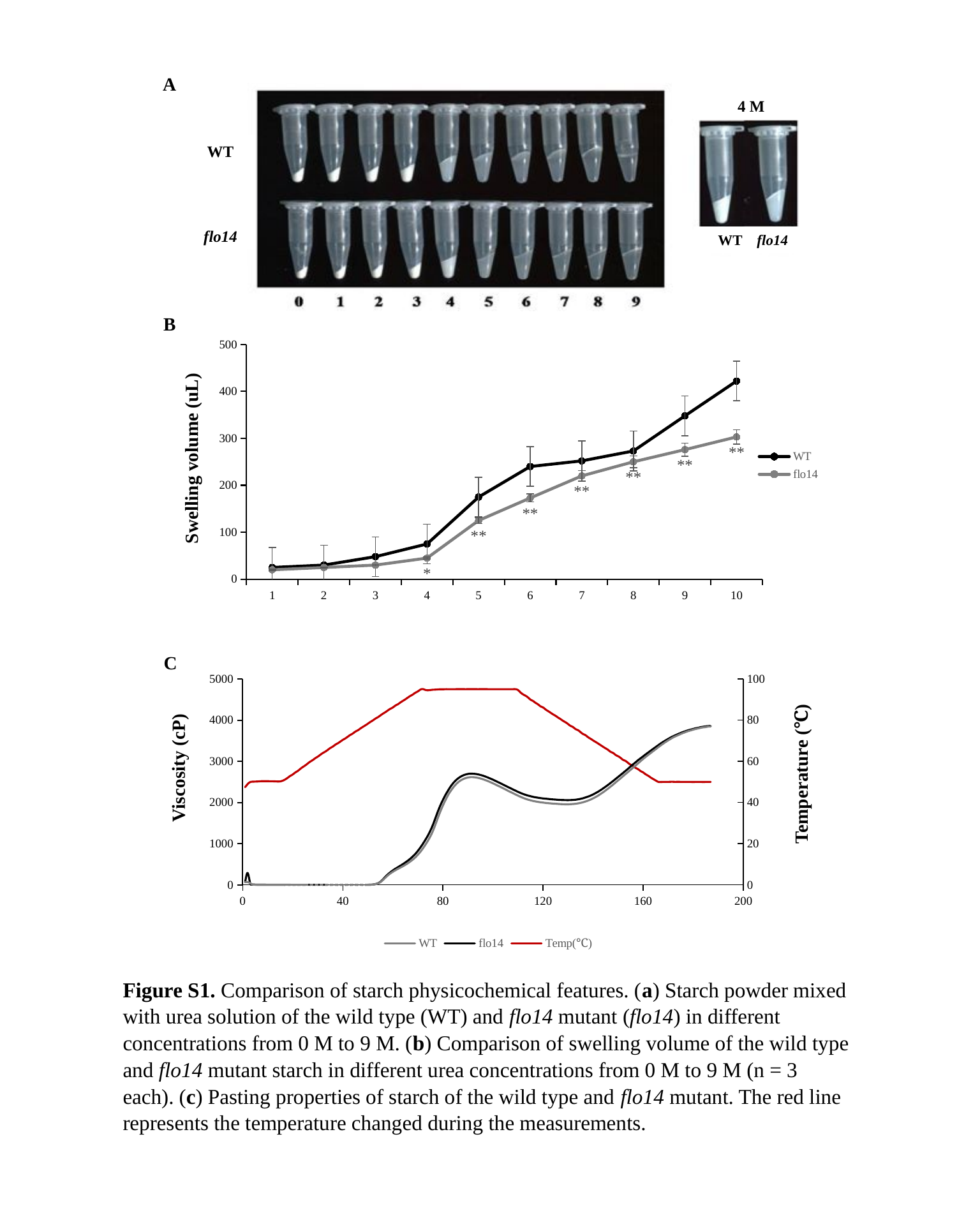

A
4 M
WT
flo14
 WT flo14
B
Swelling volume (uL)
### Chart
| Category | WT | flo14 |
|---|---|---|
### Chart
| Category | WT | flo14 | Temp(℃) |
|---|---|---|---|C
Temperature (℃)
Viscosity (cP)
Figure S1. Comparison of starch physicochemical features. (a) Starch powder mixed with urea solution of the wild type (WT) and flo14 mutant (flo14) in different concentrations from 0 M to 9 M. (b) Comparison of swelling volume of the wild type and flo14 mutant starch in different urea concentrations from 0 M to 9 M (n = 3 each). (c) Pasting properties of starch of the wild type and flo14 mutant. The red line represents the temperature changed during the measurements.
