# Supplementary material for: Lose-of-Function of a Rice Nucleolus-Localized Pentatricopeptide Repeat Protein Is Responsible for the floury endosperm14 Mutant Phenotypes
Source: Rice (N Y). 2019 Dec 30;12:100. doi: 10.1186/s12284-019-0359-x (PMC6937366; doi:10.1186/s12284-019-0359-x)
Supplement: Supplementary file 2 — Additional file 2: Table S1. The genetic analysis of flo14 mutant. [file 12284_2019_359_MOESM2_ESM.docx]

**Additional file 1**

**Table S1.** The genetic analysis of *flo14* mutant

| **Endosperm phenotype** | **Number** | **Theory** | **χ2** | **χ2**  **(0.05,1)** |
| --- | --- | --- | --- | --- |
| Normal | 188 | 193.5 | 0.048 |  |
| Floury | 67 | 61.5 | 0.164 |  |
| Total | 255 | 255 | 0.212 | 3.842 |
