# Supplementary material for: Lose-of-Function of a Rice Nucleolus-Localized Pentatricopeptide Repeat Protein Is Responsible for the floury endosperm14 Mutant Phenotypes
Source: Rice (N Y). 2019 Dec 30;12:100. doi: 10.1186/s12284-019-0359-x (PMC6937366; doi:10.1186/s12284-019-0359-x)
Supplement: Supplementary file 3 — Additional file 3: Figure S2. Young seedlings of wild type and the complementation of the flo14 mutant. [file 12284_2019_359_MOESM3_ESM.pptx]

## Slide 1
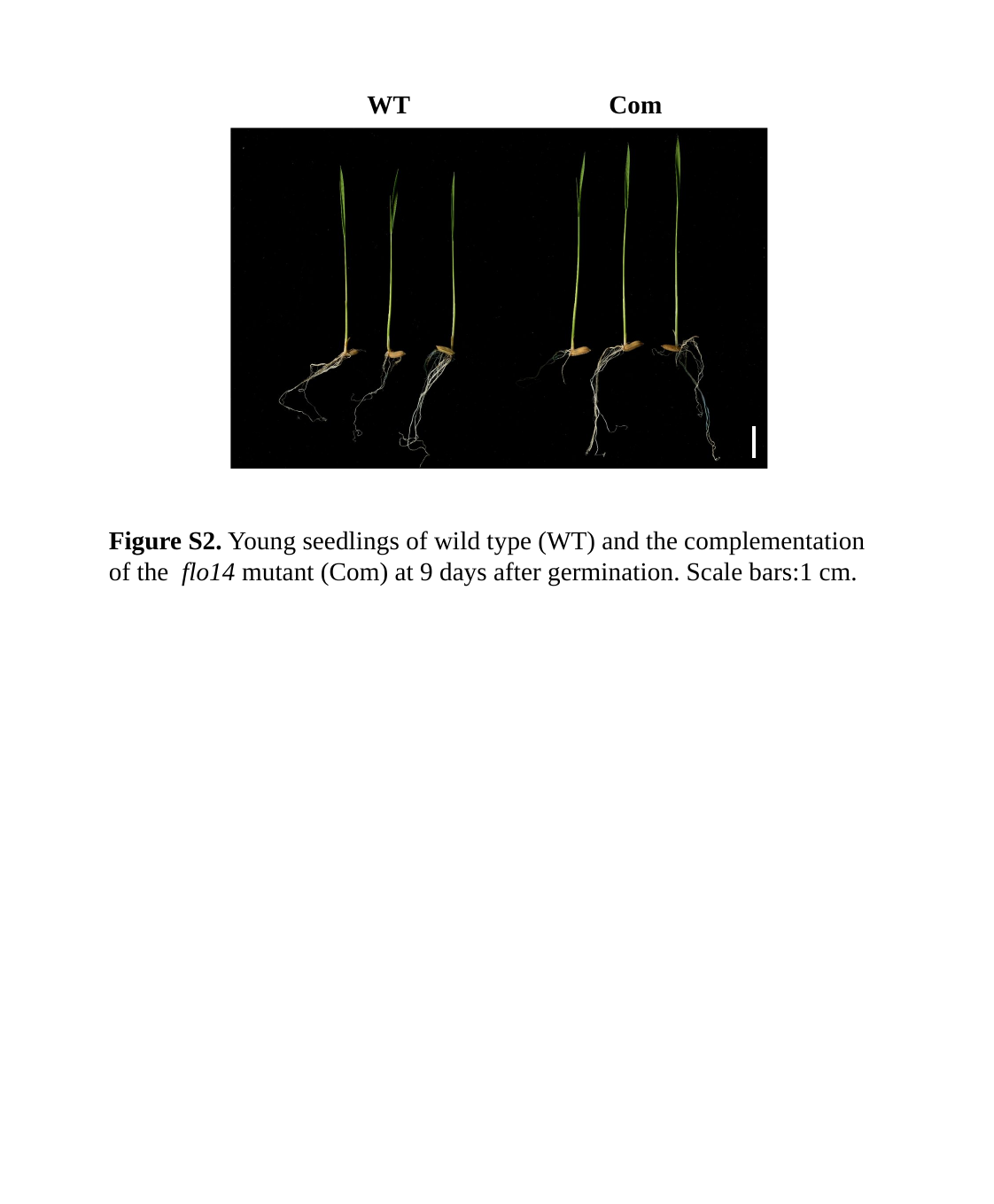

WT Com
Figure S2. Young seedlings of wild type (WT) and the complementation of the flo14 mutant (Com) at 9 days after germination. Scale bars:1 cm.
