# Supplementary material for: Lose-of-Function of a Rice Nucleolus-Localized Pentatricopeptide Repeat Protein Is Responsible for the floury endosperm14 Mutant Phenotypes
Source: Rice (N Y). 2019 Dec 30;12:100. doi: 10.1186/s12284-019-0359-x (PMC6937366; doi:10.1186/s12284-019-0359-x)
Supplement: Supplementary file 4 — Additional file 4: Figure S3. Comparison of amino acid sequences of the Os03g0728200 protein with its close species. [file 12284_2019_359_MOESM4_ESM.pptx]

## Slide 1
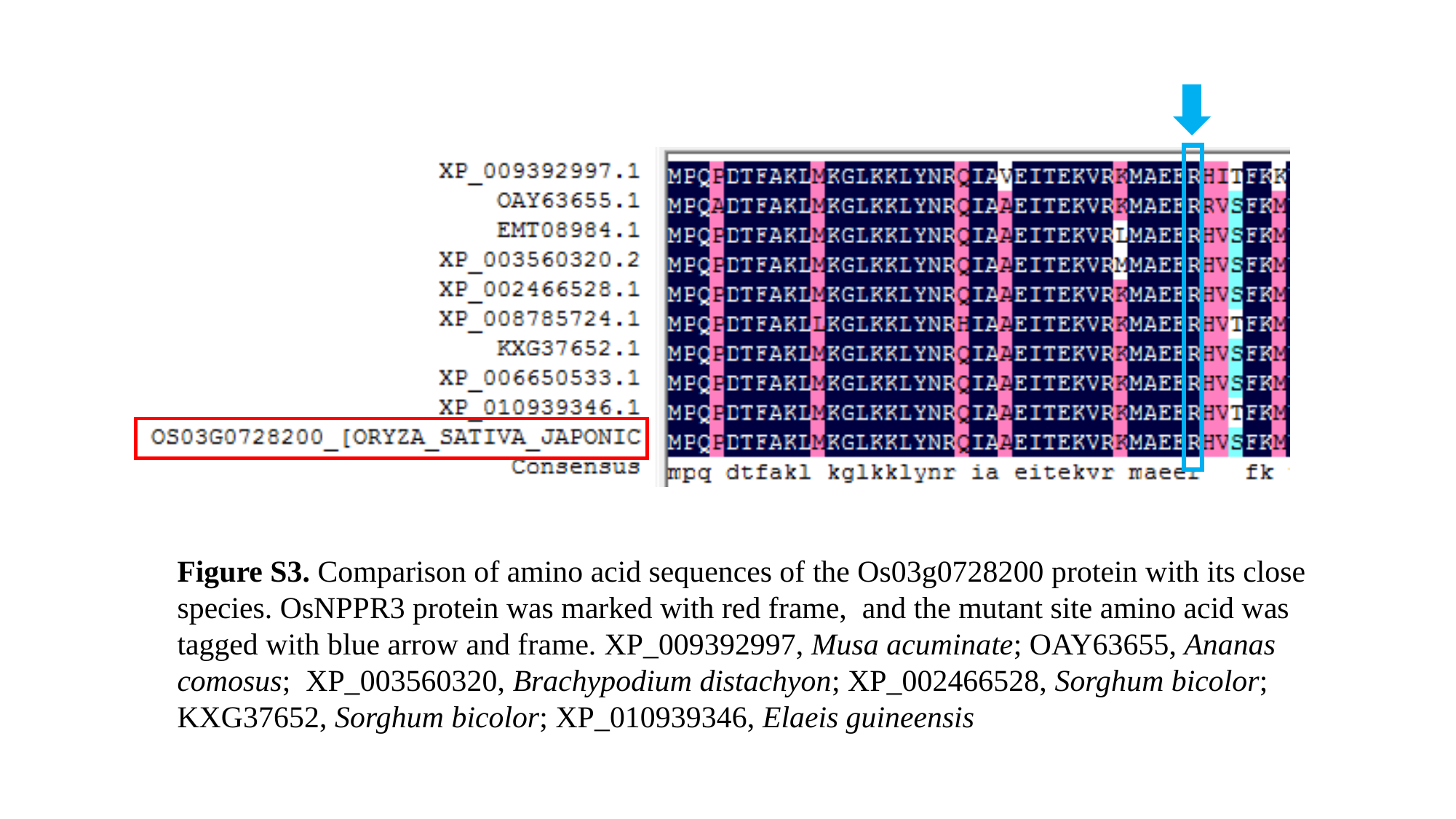

Figure S3. Comparison of amino acid sequences of the Os03g0728200 protein with its close species. OsNPPR3 protein was marked with red frame, and the mutant site amino acid was tagged with blue arrow and frame. XP_009392997, Musa acuminate; OAY63655, Ananas comosus; XP_003560320, Brachypodium distachyon; XP_002466528, Sorghum bicolor; KXG37652, Sorghum bicolor; XP_010939346, Elaeis guineensis
