# Supplementary material for: Lose-of-Function of a Rice Nucleolus-Localized Pentatricopeptide Repeat Protein Is Responsible for the floury endosperm14 Mutant Phenotypes
Source: Rice (N Y). 2019 Dec 30;12:100. doi: 10.1186/s12284-019-0359-x (PMC6937366; doi:10.1186/s12284-019-0359-x)
Supplement: Supplementary file 5 — Additional file 5: Table S2. Markers used for OsNPPR3 mapping in this study. [file 12284_2019_359_MOESM5_ESM.docx]

**Additional file 2**

**Table S2.** Markers used for *flo14* mapping in this study

| **Makers name** | **Forward primer (5’-3’)** | **Reverse primer (5’-3’)** |
| --- | --- | --- |
| FY3-0 | AGGAGAAGAGGAATCTTTGC | CGATCGAGAGCTACTATTGC |
| YYF3-1 | GAAAGGAACGGTGGATATGA | GAGATTGGGAAAGGGCAG |
| FY3-3 | GTTACGCTGCTTCTTTTTCA | ATGCACAGCCATTAACTGTA |
| FY3-6 | CGAGTCAAATTAGCCACGT | CATTCTCCTCCTTGAACCAA |
| FY3-9 | CTTCTCCTACTGCTGCTTC | TTACCCAGTCTCGAGGTAG |
