# Supplementary material for: Lose-of-Function of a Rice Nucleolus-Localized Pentatricopeptide Repeat Protein Is Responsible for the floury endosperm14 Mutant Phenotypes
Source: Rice (N Y). 2019 Dec 30;12:100. doi: 10.1186/s12284-019-0359-x (PMC6937366; doi:10.1186/s12284-019-0359-x)
Supplement: Supplementary file 6 — Additional file 6: Table S3. Primers used for vector construction. [file 12284_2019_359_MOESM6_ESM.docx]

**Additional file 3**

**Table S3.** Primers used for vector construction

| **Primer name** | **Forward primer (5’- 3’)** | **Reverse primer (5’- 3’)** |
| --- | --- | --- |
| *flo14*-1309 | TTACTTCTGCACTAGGTACCATGCGGCGCCTCCTCTCCGC | GAATTCCCGGGGATCCTTATTCATCATCAAAATCAT |
| *flo14-*CRISPER/Cas9 | GGCATCTTTGTTGCACCCCAGTAT | AAACATACTGGGGTGCAACAAAGA |
| *flo14-*DL | TGCACTGTACGCAAAGAAACACG | ACCAGCGTCAAGCATGTCCAAG |
| *flo14-*promoter-GUS | GAATTCCCGGGGATCCGCTGCGATGTTTATCCCTT | GGCCAGTGCCAAGCTTGGTGGGCTGGACTGTGGCAG |
| *flo14-*GFP | CGGAGCTAGCTCTAGA ATGCGGCGCCTCCTCTCCGC | TGCTCACCATGGATCC TTCATCATCAAAATCAT |
