# Supplementary material for: Lose-of-Function of a Rice Nucleolus-Localized Pentatricopeptide Repeat Protein Is Responsible for the floury endosperm14 Mutant Phenotypes
Source: Rice (N Y). 2019 Dec 30;12:100. doi: 10.1186/s12284-019-0359-x (PMC6937366; doi:10.1186/s12284-019-0359-x)
Supplement: Supplementary file 10 — Additional file 10: Table S4. Primers used for gene expression associated with starch synthesis. [file 12284_2019_359_MOESM10_ESM.docx]

**Additional file 4**

**Table S4.** Primers used for gene expression associated with starch synthesis (Ohdan et al., 2005)

| **Marker’ s name** | **Forward Primer (5’-3’)** | **Reverse Primer (5’-3’)** |
| --- | --- | --- |
| *AGPL 1* | CATCAAGGACGGGAAGGTCA | ACTTCACTCGGGGCAGCTTA |
| *AGPL 2* | CTGAGGAAGAGGTGCTTTGG | TCTTTCGGGAGGATTGTGTC |
| *AGPS 2b* | AACAATCGAAGCGCGAGAAA | GCCTGTAGTTAACACCCAGA |
| *BE I* | TGGCCATGGAAGAGTTGGC | CAGAAGCAACTGCTCCACC |
| *BE IIa* | GCCAATGCCAGGAAGATGA | GCGCAACATAGGATGGGTTT |
| *BE IIb* | ATGCTAGAGTTTGACCGC | AGTGTGATGGATCCTGCC |
| *GBSS I* | AACGTGGCTGCTCCTTGAA | TTGGCAATAAGCCACACACA |
| *GBSS II* | AGGCATCGAGGGTGAGGAG | CCATCTGGCCCACATCTCTA |
| *ISA I* | TGCTCAGCTACTCCTCCATCATC | AGGACCGCACAACTTCAACATA |
| *ISA II* | TAGAGGTCCTCTTGGAGG | AATCAGCTTCTGAGTCACCG |
| *PHOL* | TTGGCAGGAAGGTTTCGCT | CGAAGCCTGAAGTGAACTTGCT |
| *PPDKB* | CATGCACTGTTCGAGGAGAA | GGGAAATGGCTCTCCCTTAG |
| *PUL* | ACCTTTCTTCCATGCTGG | CAAAGGTCTGAAAGATGGG |
| *SS I* | *GGGCCTTCATGGATCAACC* | CCGCTTCAAGCATCCTCATC |
| *SS IIa* | GCTTCCGGTTTGTGTGTTCA | CTTAATACTCCCTCAACTCCACCAT |
| *SS IIIa* | GCCTGCCCTGGACTACATTG | GCAAACATATGTACACGGTTCTGG |
| *SS IIIb* | ATTCCGCTCGCAAGAACTGA | CAACCGCAGGATAACGGAAA |
| *SS IVb* | ATGCAGGAAGCCGAGATGTT | ACGACAATGGGTGCCAAGAT |
| *SUS 4* | ATGCAGGAAGCCGAGATGTT | ACGACAATGGGTGCCAAGAT |
| *UGPase I* | CAAGTACACCACAAGACCAGCAA | CGTCCAACAGCGAATCCAAT |
| *Actin* | CCCTCCTGAAAGGAAGTACAGTGT | GTCCGAAGAATTAGAAGCATTTCCC |
