# Supplementary material for: Lose-of-Function of a Rice Nucleolus-Localized Pentatricopeptide Repeat Protein Is Responsible for the floury endosperm14 Mutant Phenotypes
Source: Rice (N Y). 2019 Dec 30;12:100. doi: 10.1186/s12284-019-0359-x (PMC6937366; doi:10.1186/s12284-019-0359-x)
Supplement: Supplementary file 11 — Additional file 11: Table S5. Primers for gene expression in mitochondria. [file 12284_2019_359_MOESM11_ESM.docx]

**Additional file 5**

**Table S5.** Primers for gene expression in mitochondria

| **Primer name** | **Forward primer (5’- 3’)** | **Reverse primer (5’ - 3’)** |
| --- | --- | --- |
| *rpl 2* | CCAAGAGCTTGGACGCACA | GAAGGTCTACCTCCTTTCGT |
| *rpl 16* | GCGGAAATAGCTAGAACTGA | CCACTAACCAATTACGTTACG |
| *rpl 7* | GGGACTTTGATGGTGAGC | CTTTCGGTAAGCATCCAGTA |
| *rps 2* | CGAAATAGCTCAGTTCGAGA | GTAGCGCTACAGATTGAAGT |
| *rps 13* | GTAGATTCCAGCCGAGAAGA | TCCGAATTAGCTTGCGAGCA |
| *ATPB* | AGAGCCGCTAAACGATCGGA | CTAATTAATCTCCTTCGCAGTT |
| *NDH 4* | GATTGACTGTTGTCAACTAATC | CTGATATGCTGCCTTGATCT |
| *NDH 9* | CTGCTTAGAGCAAGAAGCG | CCACATATCCACTCAGAGGA |
| *COX I* | GACGTTGATACGCGTGCCT | GATAGCTGGAAGTTCTCCAA |
| *Cyt c* | CCAACTCCGAGCAATCTTAG | GATCACTGATCAGGTGTGAT |
| *ccmb* | CCAGCCGTCGAAGTGAATGA | TCCATGACTTGGCCATTCAA |
| *ccmFn* | GGCTTTGGGTTATGTAGATC | GCCTCCTGCTTCATCTGGTA |
| *ccmFc* | GGTCCAACTACAGAACTTCT | CTTCAAGCCCGATTTCAGGT |
| Actin | CCCTCCTGAAAGGAAGTACAGTGT | GTCCGAAGAATTAGAAGCATTTCC |
