# Supplementary material for: Lose-of-Function of a Rice Nucleolus-Localized Pentatricopeptide Repeat Protein Is Responsible for the floury endosperm14 Mutant Phenotypes
Source: Rice (N Y). 2019 Dec 30;12:100. doi: 10.1186/s12284-019-0359-x (PMC6937366; doi:10.1186/s12284-019-0359-x)
Supplement: Supplementary file 12 — Additional file 12: Table S6. Primers for genes associated with splicing in mitochondria. [file 12284_2019_359_MOESM12_ESM.docx]

**Additional file 6**

**Table S6.** Primers for genes associated with splicing in mitochondria

| **Primer name** | **Forward Primer (5' - 3')** | **Reverse Primer (5' - 3')** |
| --- | --- | --- |
| *nad 1-1* | CCCGATCATGAGTGAATAGA | GCCATGACAATCTCACTCAA |
| *nad 1-2* | AGGAGCATTACGATCTGCAG | AAGGGAGCCATCGAAAGGT |
| *nad 2* | CCTGCTGCTCATGGTGTTTC | CCACTAGATTGAGCACCAGT |
| *nad 2-1* | GTCTGAATTTTCCACGGAAG | CACGGGTTTGCCGTAATGCT |
| *nad 2-2* | ACGATAGATGCATTCGCCAT | TAGTGCTTACCTGCAGTCCA |
| *nad 3* | GCTATGAAAGCGCCACAGTT | GGCATCCCTCTTTCCTATGT |
| *nad 4-2* | GTATGGGGTTCGAGACAAAG | CAAGTCGAGTCTTATGTCGG |
| *nad 4-1* | GATTGACTGTTGTCAACTAATC | CTGATATGCTGCCTTGATCT |
| *nad 5* | GTAGGATACTTGGCCAAAGA | GGCAAGCTCCTACAGTTCTC |
| *nad 6* | CTCGAACCACAATTCTCCTT | CCATTTCAAGGGAGGACGAC |
| *nad 7* | ATGACGACTAGGAACGGGCA | CTGTGCAGTGACAGTACCAA |
| *nad 9* | CTGCTTAGAGCAAGAAGCG | CCACATATCCACTCAGAGGA |
| *atp 1* | GGAATTCTCACCCAGAGCT | CCAGTTTGTCTGTCCCCGA |
| *atp 4* | GGGATTGAGTTCAACGGATA | CAAGCCTTCCCGAATTAGGT |
| *cox 1* | GACGTTGATACGCGTGCCT | GATAGCTGGAAGTTCTCCAA |
| *cox 2* | CAGTCTCCTTTCTAGGAGCA | TGCATTTCCGCTTCAGCTTC |
| *cox 3-1* | GAAGAGCCTCCTTCTTTACC | CCACTTATTCGTTCCCTTCT |
| *cox 3-2* | CATAGTCTCGGAGGTTATGT | CCACTTATTCGTTCCCTTCT |
| *ccmFc 1* | GGTCCAACTACAGAACTTCT | CTTCAAGCCCGATTTCAGGT |
| *ccmFc 2* | GCTCTCGCCTTACCAACGA | GCAATTATGAACGAAACTTTCTC |
| *rpl 2* | CCAAGAGCTTGGACGCACA | GAAGGTCTACCTCCTTTCGT |
| *rpl 5* | CGTCAGGATCTGTTGCTCAA | CCGAAGTGACAATAGTCACA |
| *rps 2* | CGAAATAGCTCAGTTCGAGA | GTAGCGCTACAGATTGAAGT |
| *rps 3* | GGTAAGACTTGATCTGAATCG | CGACGGGGTCGAAATGCAT |
| *rps 13* | GTAGATTCCAGCCGAGAAGA | TCCGAATTAGCTTGCGAGCA |
| *orf X* | CTCATTGAATGGAATTTCGCA | GTCCAGCCCTCTTCACGAA |
| *cyt b* | CCAACTCCGAGCAATCTTAG | GATCACTGATCAGGTGTGAT |
| *UBQ* | TGGTCGTACCACAGGTATTG | CCACATCTGCTGGAATGTGCTG |
